# Extracorporeal CO_2_ removal for stable hypercapnic COPD: is it really worth it?

**DOI:** 10.1136/thoraxjnl-2020-215259

**Published:** 2020-08-05

**Authors:** Marieke L Duiverman, Peter J Wijkstra

**Affiliations:** 1 Pulmonary Diseases/Home Mechanical Ventilation, University of Groningen, University Medical Center Groningen, Groningen, Groningen, Netherlands; 2 Pulmonary Diseases, University of Groningen, University Medical Center Groningen, Groningen, Groningen, Netherlands

**Keywords:** COPD ÀÜ Mechanisms, non invasive ventilation

Long-term nocturnal non-invasive ventilation (NIV) is increasingly being applied in patients with chronic obstructive pulmonary disease (COPD) with chronic hypercapnic respiratory failure. Studies that have shown improvement in clinical and patient-related outcome measures have used a mode of ventilation aimed at a substantial reduction in carbon dioxide (CO_2_) levels.^1^ Therefore, it might be suggested that CO_2_ reduction is a causal factor for improvement in clinical outcomes such as improvement in symptoms, health-related quality of life (HRQoL) and survival.

In line with this theory, in the linked paper, Pisani *et al* present a proof-of-concept study of extracorporeal CO_2_ removal (ECCO_2_R) in patients with COPD with chronic hypercapnia unresponsive to NIV.^2^ Although this technology has been investigated in patients with COPD with acute hypercapnic respiratory failure,^3^ the study of Pisani *et al* is the first study in stable patients with COPD with chronic hypercapnia. In this small study in 10 patients with COPD with variable COPD severity (FEV_1_ ranging from 18% to 55% of predicted) and persistent hypercapnia of varying degrees (arterial carbon dioxide tension (PaCO_2_) ranging from 51.7 to 89.3 mm Hg), they showed that ECCO_2_R was safe. However, the planned 24 hours ECCO_2_R could only be completed in 6 out of 10 patients. ECCO_2_R reduced PaCO_2_ by 23%–47% and, in the patients that completed the session, this sustained for 2–4 days following ECCO_2_R interruption. Although this is an interesting concept, probably providing an alternative for NIV in patients who do not benefit from NIV or experience severe side effects, important discussion points need to be raised.

First, what is the aim of treatment in advanced patients with COPD with chronic hypercapnic respiratory failure? If we ask our patients, they do not insist on substantial CO_2_ reduction; instead they would be helped by symptom reduction, improvement in HRQoL, exacerbation reduction and improved survival. Moreover, the relationship between CO_2_ reduction and these patient-related outcomes is unclear and inconsistently shown in literature.^4^ Therefore, it is questionable whether with a therapy directed to CO_2_ reduction, similar or even better effects can be reached compared with a therapy that also influences sleep, sputum clearance, breathing patterns, ventilation-perfusion matching and lung function. Pisani *et al* do not provide any preliminary evidence of benefit in terms of patient related outcomes, which is a pity as, although it is a proof-of-concept study, patients would have been very well able to rate at least comfort and dyspnoea during and after ECCO_2_R.

We should not forget that ECCO_2_R is invasive and requires catheters to be inserted and 24 hours of ‘respiratory dialysis’ on an experienced high care unit, which is not the preferred environment for chronic severely disabled patients. In fact, nocturnal NIV for chronic respiratory failure is increasingly being offered (initiated and followed) completely at home to confine with patient wishes, and relief the burden of increasing patient numbers placed on the healthcare system.^5^ In addition, the authors showed that in 4 out of 10 patients, ECCO_2_R could not be maintained for the planned 24 hours due to different technical reasons. Conceptually, ‘CO_2_ dialysis’ can also be achieved by other means. Already 15 years ago, Diaz *et al* showed that with intermittent daytime NIV, not only CO_2_ levels, but also exercise capacity and dyspnoea improved substantially.^6^ Also, respiratory stimulant drugs, such as acetazolamide, can improve gas exchange . However, while inspiratory muscle effort is reduced by both NIV, that assists the respiratory muscles, and ECCO_2_R, with which less minute ventilation is spent to remove the produced CO_2_, with respiratory stimulant drugs, inspiratory muscle effort is expected to increase. In fact, studies on acetazolamide have shown improved oxygenation with a small fall in PaCO_2_ (3–7 mm Hg), but without positive (or even negative) effect on dyspnoea or HRQoL. Also, respiratory stimulant drugs are not effective or even harmful in patients with very severe COPD who simply cannot increase their ventilation, are not always well tolerated, and side effects might be serious and often unpredictable.^7^

Second, key to success, both for NIV and ECCO_2_R, is probably better patient selection. In COPD, it is hypothesised that chronic hypercapnia ensues once patients adopt a breathing pattern with low tidal volumes and high respiratory rate. Patients adopt this pattern to ensure that their respiratory muscles are not becoming fatigued in the context of detrimental respiratory mechanics.^8^ However, chronic hypercapnia not always develops with advanced COPD or, the other way around, might develop in patients with relative mild lung function derangements. Especially in the more obese patients, we hypothesise that a combination of disordered lung mechanics and a reduced respiratory drive contribute to nocturnal hypoventilation and the consecutive chronic daytime hypercapnia. ECCO_2_R would be most helpful in patients who need (only) resetting of their respiratory drive while having enough ventilatory capacity by themselves to change their breathing pattern during daytime. NIV would be more helpful if lung mechanics, sleep and sputum clearance needs to be supported too. In the paper of Pisani *et al* selection of patients was unfortunately not so specific, as they included patients based on daytime PaCO_2_ only. The inclusion criterion of less than 5% improvement of daytime CO_2_ with chronic NIV might reflect an inability of NIV to reduce CO_2_ but might also be effective nocturnal NIV with a fast increase at daytime in severely ventilatory limited patients. Obviously, for this proof of concept study, a wide variety of patients with COPD, with probably different underlying pathophysiology of chronic hypercapnia, was included.

To conclude, Pisani *et al* showed that ECCO_2_R is a safe treatment with effect on CO_2_ reduction. It would be exciting to see further studies with a carefully selected and characterised group of patients, preferably including measures of lung mechanics and ventilatory drive, investigating mechanisms of response and, most importantly, patient-related outcome measures, comparing it to daytime or nocturnal NIV. Taking this into account, maybe in the future, with advances in the technology, ECCO_2_R would gain its place in a selected group of advanced stable COPD.

## References

[1] Windisch W, Storre JH, Köhnlein T Nocturnal non-invasive positive pressure ventilation for COPD. Expert Rev Respir Med 2015;9:295–308. 10.1586/17476348.2015.1035260 25896807

[2] Pisani L, Nava S, Desiderio E, et al Extra-Corporeal CO2 removal (ECCO2R) in stable COPD patients with chronic hypercapnia: a proof of concept study. Thorax 2020;75:897–900. 32759384 10.1136/thoraxjnl-2020-214744

[3] Alessandri F, Pugliese F, Mascia L, et al Intermittent extracorporeal CO2 removal in chronic obstructive pulmonary disease patients. Curr Opin Crit Care 2018;24:29–34. 10.1097/MCC.0000000000000471 29135616

[4] Raveling T, Bladder G, Vonk J, et al Improvement in hypercapnia does not predict survival in COPD patients on chronic noninvasive ventilation. Int J Chron Obstruct Pulmon Dis 2018;13:3625–34. 10.2147/COPD.S169951 30464445 PMC6219270

[5] Duiverman ML, Vonk JM, Bladder G, et al Home initiation of chronic non-invasive ventilation in COPD patients with chronic hypercapnic respiratory failure: a randomised controlled trial. Thorax 2020;75:244–52. 10.1136/thoraxjnl-2019-213303 31484786 PMC7063397

[6] Díaz O, Bégin P, Andresen M, et al Physiological and clinical effects of diurnal noninvasive ventilation in hypercapnic COPD. Eur Respir J 2005;26:1016–23. 10.1183/09031936.05.00033905 16319330

[7] Adamson R, Swenson ER Acetazolamide use in severe chronic obstructive pulmonary disease. Pros and Cons. Ann Am Thorac Soc 2017;14:1086–93. 28622013 10.1513/AnnalsATS.201701-016FR

[8] Gorini M, Misuri G, Corrado A, et al Breathing pattern and carbon dioxide retention in severe chronic obstructive pulmonary disease. Thorax 1996;51:677–83. 10.1136/thx.51.7.677 8882072 PMC472488

